# The Finnish genetic heritage in 2022 – from diagnosis to translational research

**DOI:** 10.1242/dmm.049490

**Published:** 2022-10-26

**Authors:** Johanna Uusimaa, Johannes Kettunen, Teppo Varilo, Irma Järvelä, Jukka Kallijärvi, Helena Kääriäinen, Minna Laine, Risto Lapatto, Päivi Myllynen, Harri Niinikoski, Elisa Rahikkala, Anu Suomalainen, Ritva Tikkanen, Henna Tyynismaa, Päivi Vieira, Tomas Zarybnicky, Petra Sipilä, Satu Kuure, Reetta Hinttala

**Affiliations:** ^1^ Children and Adolescents, Oulu University Hospital, 90029 Oulu, Finland; ^2^ Research Unit of Clinical Medicine and Medical Research Center, Oulu University Hospital and University of Oulu, 90014 Oulu, Finland; ^3^ Computational Medicine, Center for Life Course Health Research, University of Oulu, 90014 Oulu, Finland; ^4^ Department of Public Health and Welfare, Finnish Institute for Health and Welfare, 00271 Helsinki, Finland; ^5^ Biocenter Oulu, University of Oulu, 90014 Oulu, Finland; ^5^ Department of Medical Genetics, University of Helsinki, P.O. Box 720, 00251 Helsinki, Finland; ^6^ Department of Medical Genetics, University of Helsinki, 00251 Helsinki, Finland; ^7^ Folkhälsan Institute of Genetics, Folkhälsan Research Center, 00014 Helsinki, Finland; ^8^ Stem Cells and Metabolism Research Program, Faculty of Medicine, University of Helsinki, 00014 Helsinki, Finland; ^9^ Department of Pediatric Neurology, Helsinki University Hospital and University of Helsinki, 00029 Helsinki, Finland; ^10^ Children's Hospital, University of Helsinki and Helsinki University Central Hospital, 00029 Helsinki, Finland; ^11^ Department of Clinical Chemistry, Cancer and Translational Medicine Research Unit, Medical Research Center, University of Oulu and Northern Finland Laboratory Centre NordLab, Oulu University Hospital, 90029 Oulu, Finland; ^12^ Research Centre for Integrative Physiology and Pharmacology, Institute of Biomedicine, University of Turku, 20014 Turku, Finland; ^13^ Research Centre of Applied and Preventive Cardiovascular Medicine, University of Turku, 20014 Turku, Finland; ^14^ Centre for Population Health Research, University of Turku and Turku University Hospital, 20014 Turku, Finland; ^15^ Department of Pediatrics, Turku University Hospital, 20014 Turku, Finland; ^16^ Department of Clinical Genetics, Oulu University Hospital, 90029 Oulu, Finland; ^17^ HUS Diagnostics, Helsinki University Hospital, 00014 Helsinki, Finland; ^18^ Institute of Biochemistry, Medical Faculty, University of Giessen, D-35392 Giessen, Germany; ^19^ Neuroscience Center, Helsinki Institute of Life Science, University of Helsinki, 00014 Helsinki, Finland; ^20^ Helsinki Institute of Life Science, University of Helsinki, 00014 Helsinki, Finland; ^21^ Turku Center for Disease Modeling, Institute of Biomedicine, University of Turku, 20014 Turku, Finland; ^22^ GM-Unit, Laboratory Animal Center, Helsinki Institute of Life Science, University of Helsinki, 00014 Helsinki, Finland

**Keywords:** FinnGen, Finnish disease heritage, Big data, Monogenic disorders, Population isolate, Rare disease

## Abstract

Isolated populations have been valuable for the discovery of rare monogenic diseases and their causative genetic variants. Finnish disease heritage (FDH) is an example of a group of hereditary monogenic disorders caused by single major, usually autosomal-recessive, variants enriched in the population due to several past genetic drift events. Interestingly, distinct subpopulations have remained in Finland and have maintained their unique genetic repertoire. Thus, FDH diseases have persisted, facilitating vigorous research on the underlying molecular mechanisms and development of treatment options. This Review summarizes the current status of FDH, including the most recently discovered FDH disorders, and introduces a set of other recently identified diseases that share common features with the traditional FDH diseases. The Review also discusses a new era for population-based studies, which combine various forms of big data to identify novel genotype–phenotype associations behind more complex conditions, as exemplified here by the FinnGen project. In addition to the pathogenic variants with an unequivocal causative role in the disease phenotype, several risk alleles that correlate with certain phenotypic features have been identified among the Finns, further emphasizing the broad value of studying genetically isolated populations.

## Teaser

**Summary:** This Review discusses the unique enrichment of variants causing Finnish disease heritage disorders and the discovery of novel genotype–phenotype associations underlying monogenic and common complex diseases.

## The Finns as an isolated population

Population isolates are defined as subpopulations originally derived from a small group of individuals who became isolated because of a certain founding event, e.g. settlement of a new territory, and have remained isolated for several generations. Similar to this founder effect, the bottleneck phenomenon, in which few survivors remain from a larger population after e.g. famine, war or epidemics has a major effect on the genetic drift of an isolated population. In both cases, genetic drift favors some genetic variants and eliminates others.

Finland has a population history characterized by features of founder effect, bottlenecks, genetic drift and isolation. Fairly small founder populations, of around 20-40 families, have inhabited the relatively large country, generating internal subisolates (Norio et al., 1973; Peltonen, 1997; Martin et al., 2018; Norio, 2003b). Living next to Eastern European populations has influenced the genetic background of the Finnish population over the past generations by facilitating and maintaining isolation. Genealogical data on births, deaths, marriages and movements of the majority of the Finnish population have been archived in the church records dating back to 1640 (Peltonen et al., 1995). These have been replaced by the modern Digital and Population Data Services Agency (The Finnish Digital Agency, https://dvv.fi/en), meaning that extensive historical and contemporary records are available for research. Church records dating back to the mid-17th century and taxation documents still play a significant role in today's successful studies on genetic traits in the Finnish population, and we encourage readers to seek more information in Peltonen (1997) and Peltonen et al., (1999).

A number of deleterious genetic variants have been significantly enriched among the Finns due to relatively recent bottleneck events that have not yet undergone negative selection (Lim et al., 2014). This is reflected in the regional enrichment of certain alleles, some of them causative for the Finnish disease heritage (FDH). FDH is a group of nearly 40 rare monogenic diseases that are over-represented in Finland, with symptoms ranging from adult-onset mildly disabling to embryonically lethal (Norio, 2003a; Polvi et al., 2013). Almost one-third of the diseases cause intellectual disability, and a similar fraction present with visual defects. In addition, congenital malformations, bone disorders, hearing loss, metabolic disturbances, epileptic or deteriorating neurological diseases, blood disorders and multisystemic syndromes are represented among FDH (Table 1). Conversely, some relatively common genetic diseases, such as phenylketonuria, galactosemia and cystic fibrosis, are exceptionally rare or even absent in Finns (Perheentupa, 1995).

**Table 1.**
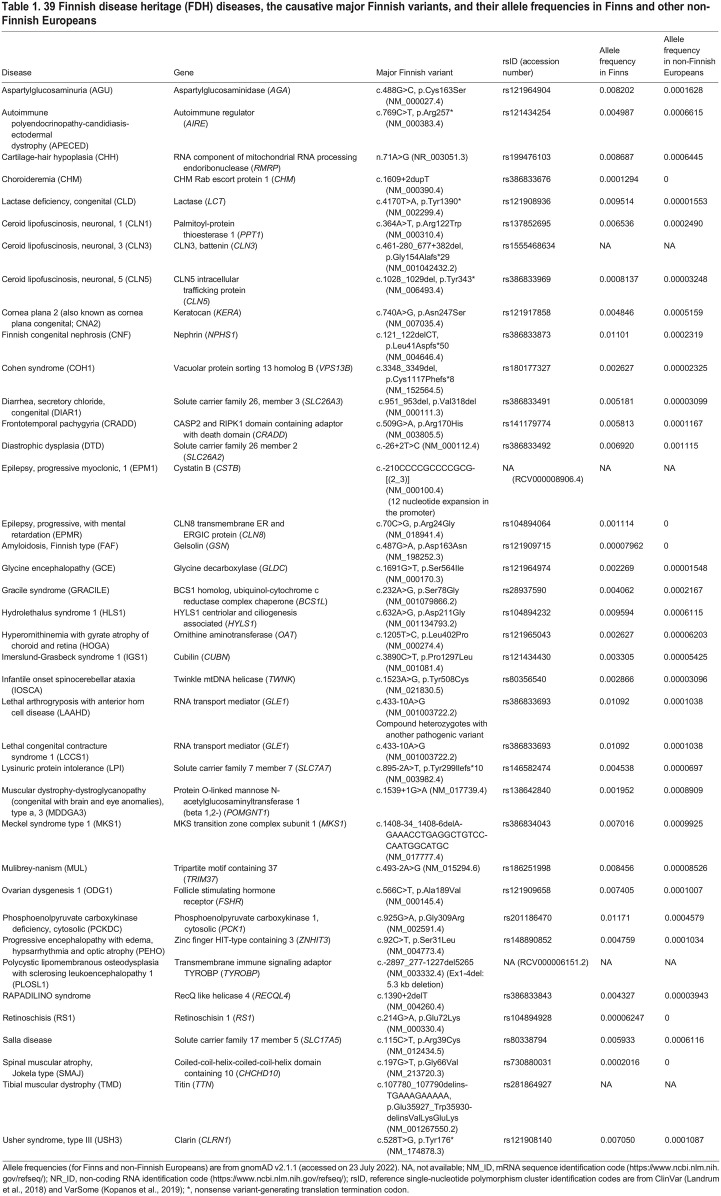
39 Finnish disease heritage (FDH) diseases, the causative major Finnish variants, and their allele frequencies in Finns and other non-Finnish Europeans

In this Review, we describe the latest achievements in FDH research on the underlying molecular mechanisms, development of treatment options and most recent discoveries of genetic diseases enriched in the Finnish population. The Review discusses the FinnGen project, which combines health care data with genetic variant data of Finnish biobank samples, expanding the value of this founder population for genetic association studies of complex diseases and quantitative traits of common diseases.

## The FDH

In the early 1960s, Dr Reijo Norio investigated patients with congenital nephrosis, conducted genealogical studies, discovered the autosomal-recessive mode of inheritance of these diseases and clarified the distribution of the disease alleles in rural Finland. In 1966, Norio's thesis outlined how founder effect, genetic drift and isolation had molded the Finnish gene pool towards both over- and under-representation of some genetic diseases (Norio, 1966). In 1973, the Finnish pediatricians Reijo Norio, Harri Nevanlinna and Jaakko Perheentupa defined the FDH to increase clinical knowledge and encourage research of these diseases, which mainly affect Finnish children (Norio et al., 1973). It soon became common practice among Finnish medical geneticists to draw pedigrees and estimate time of enrichment of all such disease-causing variants to the subisolates (Varilo, 1999). Furthermore, within the next two decades, Profs Albert de la Chapelle and Leena Peltonen and their students cloned the majority of the pathogenic genetic variants of the FDH (Polvi et al., 2013).

It was originally postulated that the FDH diseases should be more common in Finland than in the rest of the world (Norio, 1981). However, although Finnish physicians originally described many of these diseases, foreign colleagues later identified some of the diseases in other population isolates as well, and the criterion was changed to markedly over-represented in Finland (Norio, 2003a). The second criterion for inclusion in the FDH was that clinicians should detect a given disease in more than ten core families (Norio, 2003a).

**Fig. 1.**
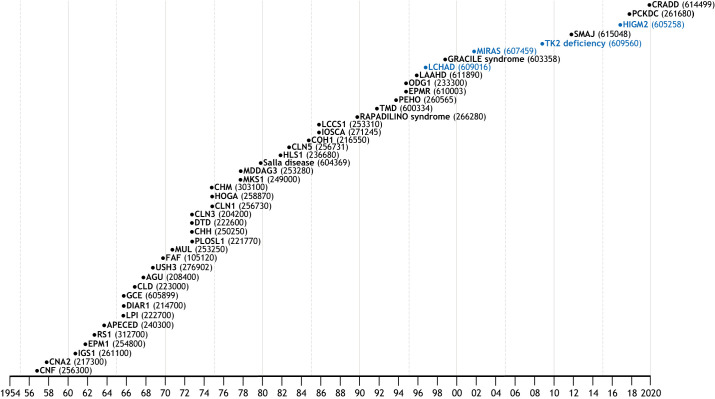
**Updated ‘Perheentupa Steps’.** This widely used traditional representation of the Finnish disease heritage (FDH) diseases organizes them chronologically based on the year in which the disease description was published. The respective Online Mendelian Inheritance in Man (OMIM) codes are in parentheses. FDH diseases are in black and candidate diseases in blue. CNF, Finnish congenital nephrosis (Ahvenainen et al., 1956); CNA2, cornea plana 2 (Forsius, 1957); IGS1, Imerslund-Grasbeck syndrome 1 (Gräsbeck et al., 1960); EPM1, epilepsy, progressive myoclonic, 1 (Harenko and Toivakka, 1961); RS1, retinoschisis 1 (Forsius et al., 1962); APECED, autoimmune polyendocrinopathy-candidiasis-ectodermal dystrophy – type 1 with or without reversible metaphyseal dysplasia (Visakorpi and Gerber, 1963); LPI, lysinuric protein intolerance (Perheentupa and Visakorpi 1965); DIAR1, diarrhea, secretory chloride, congenital (Perheentupa et al., 1965); GCE, glycine encephalopathy (Visakorpi et al., 1965); CLD, lactase deficiency, congenital (Launiala et al., 1966); AGU, aspartylglucosaminuria (Palo, 1967); USH3, Usher syndrome type 3 (Nuutila, 1968); FAF, amyloidosis, Finnish type (Meretoja, 1969); MUL, Mulibrey nanism (Perheentupa et al., 1970); PLOSL1, polycystic lipomembranous osteodysplasia with sclerosing leukoencephalopathy 1 (Hakola, 1972); CHH, cartilage-hair hypoplasia (Perheentupa, 1972); DTD, diastrophic dysplasia (Perheentupa, 1972); CLN3, ceroid lipofuscinnosis, neuronal, 3 (Santavuori, 1972); CLN1, ceroid lipofuscinosis, neuronal 1 (Santavuori et al., 1973); HOGA, hyperornithinemia with gyrate atrophy of choroid and retina (Simell and Takki, 1973); CHM, choroideremia (Takki, 1974); MKS1, Meckel syndrome type 1 (Aula et al., 1977); MDDAG3, muscular dystrophy-dystroglycanopathy (congenital with brain and eye anomalies), type a, 3 (Santavuori, et al., 1977); Salla disease (Aula et al., 1979); HLS1, hydrolethalus syndrome 1 (Salonen et al., 1981); CLN5, ceroid lipofuscinnosis, neuronal, 5 (Santavuori et al., 1982); COH1, Cohen syndrome (Norio et al., 1984); IOSCA, infantile-onset spinocerebellar ataxia (Kallio and Jauhianen, 1985); LCCS1, lethal congenital contracture syndrome 1 (Herva et al., 1985); RAPADILINO syndrome (Kaariainen et al., 1989); TMD, tibial muscular dystrophy (Udd et al., 1991); PEHO, progressive encephalopathy with edema, hypsarrhythmia and optic atrophy (Salonen et al., 1991); EPMR, epilepsy, progressive, with mental retardation (Hirvasniemi et al., 1994); ODG1, ovarian dysgenesis 1 (Aittomäki, 1994); LAAHD, lethal arthrogryposis with anterior horn cell disease (Vuopala et al., 1995); LCHAD, long-chain 3-hydroxyacyl-CoA dehydrogenase deficiency (Tyni et al., 1996); GRACILE syndrome (Fellman et al., 1998); MIRAS, mitochondrial recessive ataxia syndrome (Rantamäki et al., 2001); TK2 deficiency (Götz et al., 2008); SMAJ, spinal muscular atrophy, Jokela type (Jokela et al., 2011); HIGM2, hyper-IgM syndrome type 2 (Trotta et al., 2016); PCKDC, phosphoenolpyruvate carboxykinase deficiency, cytosolic (Vieira et al., 2017); CRADD, frontotemporal pachygyria (Polla et al., 2019). Original illustration of the steps was first used by Prof. Jaakko Perheentupa and colleagues (Norio et al., 1973).

As research has progressed, geneticists can now relatively easily ascertain whether a novel FDH candidate disease has a founder mutation (see Glossary, Box 1) and whether the allele frequency clearly differs between Finnish and other populations. Today, FDH comprises 39 diseases, with 34 autosomal-recessive, three autosomal-dominant and two X-linked ones (Table 1, Fig. 1). The clearly defined FDH offers practical advantages. First, as clinicians recognize the characteristic clinical features of FDH disorders and the causal founder mutations are known, the diseases can be diagnosed relatively easily and inexpensively with targeted variant sequencing. Second, translational research towards better understanding of the natural course of the disease and developing treatment options is ongoing. Together with the research community and systematic phenotyping conducted by the International Mouse Phenotyping Consortium (IMPC; https://www.mousephenotype.org), knockout (KO) mice for the majority of genes causing FDH have already been generated. However, as many of the disease-causing variants of FDH are missense and the knock-in mouse models expressing the Finnish major mutations are currently not available, only a few of the existing KO mouse lines recapitulate the full spectrum of FDH disease symptoms (Table 2) (Zárybnický et al., 2021). Thus, strategies for better *in vivo* and *in vitro* disease models that faithfully genocopy disease-specific variants and phenocopy the FDH disease symptoms are needed and constantly developed. As a result of these concerted efforts, some disease-specific biomarkers and treatment options are available today, as described later in this Review. Third, improved diagnostics and genetic counseling have offered families new options for family planning that, together with migration within and from outside the country, has decreased the number of patients with some of the most devastating diseases.

**Box 1. Glossary**

**Aspartylglucosaminidase (AGA):** lysosomal hydrolase that is involved in the degradation of N-glycosylated glycoproteins.

**BCS1 homolog, ubiquinol-cytochrome c reductase complex chaperone (*BCS1L*):** gene encoding an ATPase located in the inner mitochondrial membrane and required for the assembly of the Rieske iron-sulphur protein (RISP, UQCRFS1) subunit into mitochondrial respiratory chain complex III.

**CASP2 and RIPK1 domain containing adaptor with death domain (*CRADD*):** gene encoding an adaptor protein that is required for activation of caspase-2-mediated apoptosis.

**Coiled-coil-helix-coiled-coil-helix domain containing 10 (*CHCHD10*):** gene encoding a mitochondrial intermembrane space protein that is enriched at cristae junctions.

**DNA polymerase gamma, catalytic subunit (*POLG*):** gene encoding the catalytic subunit of the mitochondrial DNA polymerase that is responsible for replication of the mitochondrial genome.

**Founder mutation:** a genetic alteration observed with high frequency in an isolated population, in which one or several of the ancestors were carriers of the altered gene variant.

**Hypochromic anemia:** anemia in which the circulating red blood cells have decreased red color.

**Lissencephaly:** brain disorder caused by deficient neuronal migration resulting in cortical thickening and reduced gyration.

**Minor allele frequency (MAF):** frequency at which the second-most common allele occurs in a population.

**Mitochondrial trifunctional protein (MTP):** enzyme complex that consists of four α subunits encoded by *HADHA* gene with long-chain enoyl-CoA hydratase and long-chain 3-hydroxyacyl-CoA dehydrogenase activities, and four β-subunits encoded by *HADHB* gene with long-chain thiolase activity. Locates in the mitochondrial inner membrane and catalyzes the last steps in β-oxidation of long-chain fatty acids.

**Normochromic anemia:** anemia in which the concentration of hemoglobin in the red blood cells is within the standard range, but there is an insufficient number of red blood cells.

**Orotic aciduria:** condition characterized by megaloblastic anemia and orotic acid crystalluria.

**Rieske iron-sulphur protein (RISP; also known as UQCRFS1)**: a subunit of mitochondrial respiratory chain complex III.

**Solute carrier family 7 member 7 (*SLC7A7*):** gene encoding the light subunit of the dibasic amino acid transporter y+LAT-1.

**Thymidine kinase 2 (*TK2*):** gene encoding mitochondrial enzyme involved in the phosphorylation of thymidine, deoxycytidine, and deoxyuridine in the mitochondrial matrix.

**Trimethylglycine (also known as betaine):** an amino acid derivative that is involved in methylation reactions and detoxification of homocysteine, distributed under the brand name Cystadane^®^.

**Twinkle mtDNA helicase (*TWNK*):** gene encoding the replicative helicase of mitochondrial DNA.

**Table 2.**
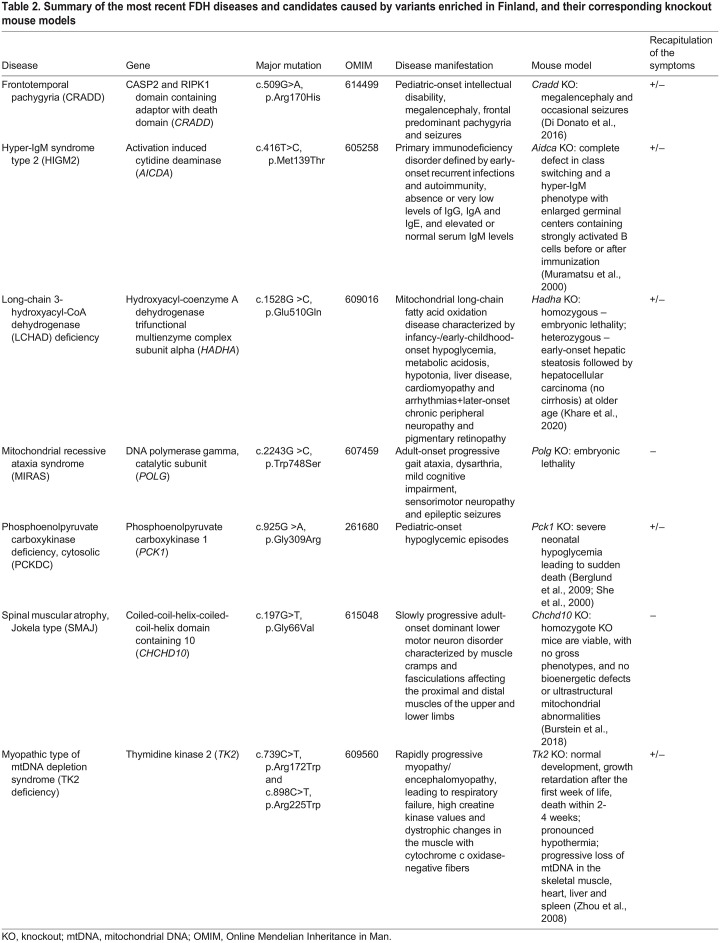
Summary of the most recent FDH diseases and candidates caused by variants enriched in Finland, and their corresponding knockout mouse models

### FDH diseases and treatment options

Over the years, a combination of basic, translational and clinical research has provided valuable information on genetic causes, molecular mechanisms and clinical manifestations of FDH diseases. In the following sections, we discuss recent advancements in understanding causative pathways and treatment options for a selection of FDH diseases: aspartylglucosaminuria (AGU), GRACILE syndrome, infantile-onset spinocerebellar ataxia (IOSCA) and lysinuric protein intolerance (LPI).

#### AGU

AGU [Online Mendelian Inheritance in Man (OMIM) 208400] is a neuropediatric lysosomal storage disease that results from pathogenic variants in the *AGA* gene (Box 1). AGA deficiency leads to the accumulation of glycoasparagine conjugates in the cells and body fluids of AGU patients, causing a slow but progressive developmental delay that manifests early in childhood, with intellectual disability only becoming evident by the age of 7-10 years (Arvio and Mononen, 2016; Goodspeed et al., 2021; Harjunen et al., 2020). In addition to developmental delay, AGU patients present with motor clumsiness, recurrent infections, behavioral problems and mild to moderate skeletal problems, as well as coarse facial features, increased risk of epilepsy and severe retardation later in life.

**Fig. 2.**
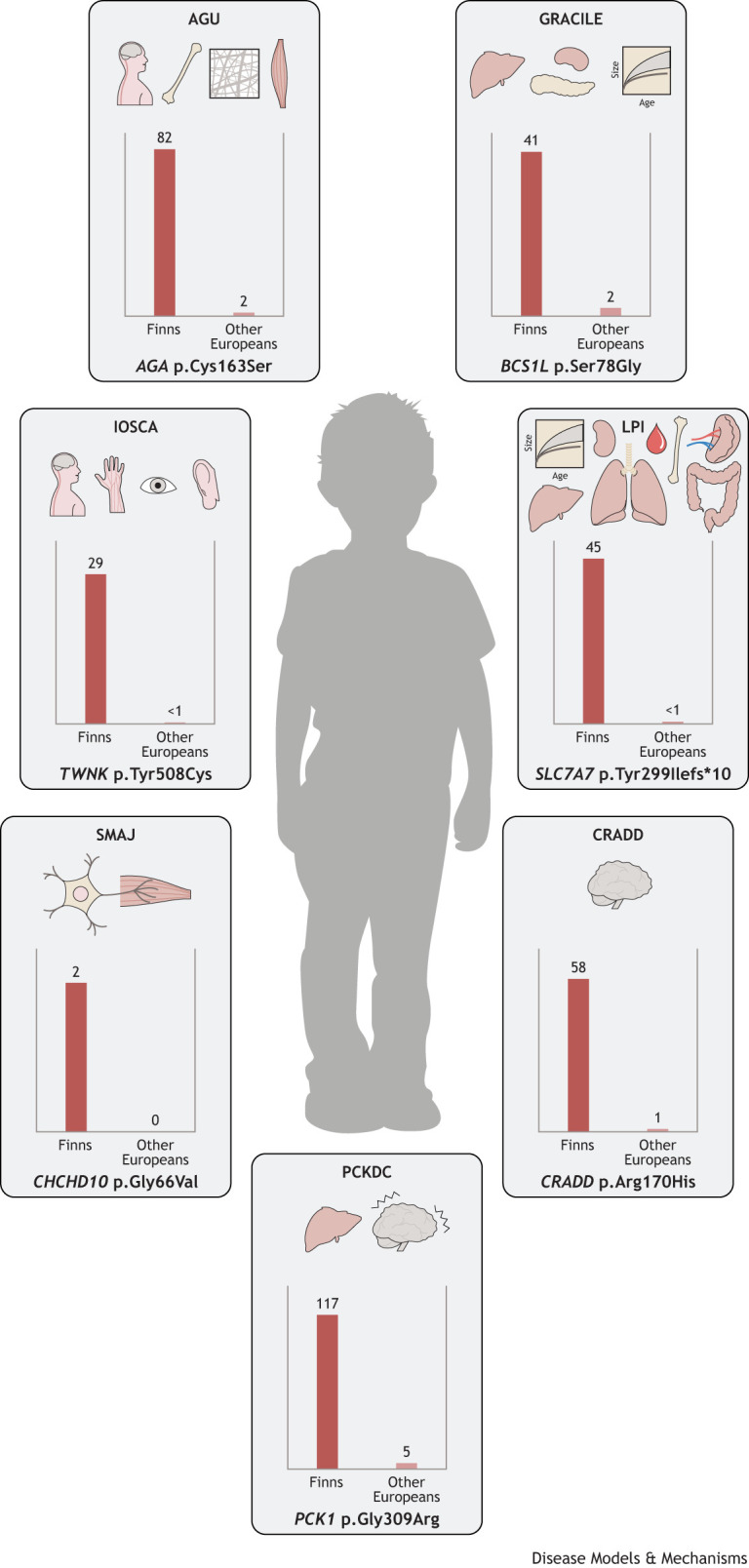
**The traditional FDH diseases with their core manifestations and estimated frequencies of major Finnish variants.** Aspartylglucosaminuria (AGU) is caused by a mutation in *AGA* and affects the central nervous system, skeleton, connective tissue and skeletal muscle. GRACILE syndrome (GRACILE) is caused by a mutation in *BCS1L* that affects the liver, kidneys and pancreas, and causes early-onset growth retardation. Infantile-onset spinocerebellar ataxia (IOSCA), which is caused by a mutation in *TWNK*, affects the central and peripheral nervous systems, eyes and hearing. Lysinuric protein intolerance (LPI), caused by a *SLC7A7* mutation, results in growth retardation and also affects the kidneys, blood, liver, spleen, skeleton, intestine and lungs. Spinal muscular atrophy, Jokela type (SMAJ) is caused by a mutation in *CHCHD10* and affects the lower motor neurons, whereas frontotemporal pachygyria (CRADD) is caused by a *CRADD* mutation and affects the brain. The metabolic disruption caused by phosphoenolpyruvate carboxykinase (PCK1) deficiency, cytosolic (PCKDC) affects the liver and causes hypoglycemic seizures. The graphs show estimated carriers per 10,000 Finns and other Europeans. Minor allele frequencies (MAFs) are based on gnomAD v2.1.1 (https://gnomad.broadinstitute.org/), accessed on 23 July 2022.

Most of the AGU patients are found in Finland, where two to three AGU patients are born every year. The majority of patients in Finland carry a specific *AGA* c.488G>C, p.Cys163Ser (NM_000027.4) variant, also known as AGU_Fin-major_, occurring either homozygously or as a compound heterozygous mutation with another pathogenic *AGA* variant (Ikonen et al., 1991a,b; Mononen et al., 1991). The minor allele frequency (MAF; Box 1) of AGU_Fin-major_ is 50 times higher in Finns than in non-Finnish Europeans (Table 1, Fig. 2) (Karczewski et al., 2020). AGU patients elsewhere in the world usually carry their own family-specific variants, with few exceptions, and over 40 pathogenic *AGA* variants are known (Saarela et al., 2001; Ikonen et al., 1991a,b; Opladen et al., 2014; Banning et al., 2016, 2018; Laitinen et al., 1997). Although AGU is currently considered a Finnish disease, genome and exome sequencing have improved the identification of AGU patients outside Finland. Improved clinical and molecular diagnostics outside Finland is very important, as it will enhance interest in therapy development and facilitate therapy accessibility for a larger number of patients.

The first AGU-causing variant was identified some 30 years ago, but major steps towards therapies have been taken only recently. Currently, there are no approved disease-specific therapies. However, several preclinical studies aiming at either variant-specific or common therapies have been carried out (Peltola et al., 1998). Very recently, adeno-associated virus (AAV)-mediated gene therapy was shown to ameliorate the disease phenotype in the *Aga* KO mouse model (Chen et al., 2021). Furthermore, enzyme replacement therapy has also been tested in the KO mouse model (Dunder et al., 2010; Kelo et al., 2005), but clinical development is complicated by difficulties in producing sufficient amounts of recombinant AGA for the treatment of patients.

Both gene and enzyme replacement therapies are suitable for most patients. However, a number of small-molecule-based variant-specific approaches have recently been developed without *in vivo* preclinical testing (Banning et al., 2016, 2018). Betaine (trimethylglycine; Box 1) can enhance the enzymatic activity of the AGU_Fin-major_ AGA variant, where loss of a disulfide bridge due to the Cys163Ser substitution results in a folding defect that is ameliorated by betaine (Banning et al., 2016). A clinical study with betaine is currently running in Finland [EU Clinical Trials Register, https://www.clinicaltrialsregister.eu/ctr-search/trial/2017-000645-48/FI (accessed on 9 July 2022)]. Furthermore, small molecules such as Amlexanox may improve translation of specific nonsense *AGA* variants, but are only suitable for a low number of patients (Banning et al., 2018). Very recently, flavonoids and methylxanthines have been identified as splicing enhancers of *AGA* pre-mRNAs that carry substitutions in the intronic splice-acceptor sites (Banning and Tikkanen, 2021). These substances show promise not only in AGU, but also in other diseases caused by similar splicing defects.

According to recent findings emerging from magnetic resonance imaging (MRI) techniques, such as susceptibility-weighted imaging, AGU children have age-dependent accumulation of iron in thalamic regions in the brain. This imaging technique can be used in diagnostics and possibly as a biomarker (Tokola et al., 2019; Sairanen et al., 2020). Although the specific molecular defect in AGU results in accumulation of undigested glycoprotein fragments, this might not be the only cellular dysfunction that causes the disease phenotype. Many lysosomal storage diseases share common pathological features, such as defects in autophagy and lysosomal signaling, or abnormal amounts of specific lipids (Platt et al., 2018). Therefore, it may be possible to develop therapies against these common pathologies that would thus be suitable for a number of diseases (Platt, 2018). In addition, combination therapies, e.g. with AAV-based gene therapy and small molecules, should also be tested, as ameliorating disease symptoms requires the treatment of both the central nervous system and the periphery.

#### GRACILE syndrome

Mitochondrial diseases as a group are the most common inborn errors of metabolism. They are caused by variants in the mitochondrial DNA (mtDNA) or in the nuclear genes encoding mitochondrial proteins and typically manifest as (cardio)myopathy or encephalopathy, but can show a wide variety of other manifestations, including visceral pathology (Hikmat et al., 2021). Variants in several genes encoding mitochondrial respiratory complex (C)III subunits or proteins assisting the assembly of its 11 subunits cause rare CIII deficiency syndromes (Banerjee et al., 2021; Fellman and Kotarsky, 2011). The phenotypic heterogeneity and pathomechanisms of CIII deficiencies remain poorly understood, and thus only supportive treatment is available. GRACILE syndrome (growth restriction, aminoaciduria, cholestasis, iron overload, lactic acidosis, early death; OMIM 603358), first described by the neonatologist Vineta Fellman in Finland, is the most severe CIII deficiency. It manifests as fetal-onset growth restriction, hepatopathy, Fanconi-type kidney tubulopathy and lactic acidosis, but lacks encephalopathy and myopathy during the short lifespan of patients, which typically ranges from a few days to a few months (Fellman et al., 1998; Rapola et al., 2002).

All known cases of GRACILE syndrome in Finland have been caused by a single missense c.232A>G, p.Ser78Gly (NM_001079866.2) variant in *BCS1L* (Box 1) (Visapää et al., 2002). The MAF of *BCS1L* c.232A>G is 19 times higher in Finns than in non-Finnish Europeans (Table 1, Fig. 2) (Karczewski et al., 2020). Some 20 other pathogenic *BCS1L* variants cause a wide spectrum of phenotypes, from severe GRACILE-like neonatal disorders to the mild Björnstad syndrome (Baker et al., 2019; Hikmat et al., 2021). These are either homozygous missense variants or compounds of a nonsense and a missense or splice site variant. No patients with homozygous nonsense variants have been identified, suggesting that full loss of BCS1L function is incompatible with life. Decreased BCS1L function leads to compromised UQCRFS1 (RISP; Box 1) subunit assembly and loss of CIII activity (Kotarsky et al., 2010). The recently elucidated structure of mouse allows precise structural modeling of the missense variants, and could eventually explain the associated clinical heterogeneity (Tang et al., 2020). The early patient studies of GRACILE syndrome were complicated by the fact that CIII deficiency generally does not manifest in fibroblasts (Kotarsky et al., 2010), and this continues to be a challenge to studies on the effects of the different pathogenic variants.

To aid mechanistic and interventional studies, Prof. Fellman's group generated a homozygous *Bcs1l^p.Ser78Gly^* knock-in mouse model. At the time, this was the only patient mutation-specific and viable animal model of CIII deficiency (Levéen et al., 2011). *Bcs1l^p.Ser78Gly^* mice are born healthy, but display growth failure and progressive liver disorder from the fourth week of age, renal tubulopathy, loss of white adipose tissue, hypoglycemia and consistent short lifespan of ∼35 days (Levéen et al., 2011; Davoudi et al., 2014; Rajendran et al., 2016). Liver pathology is characterized by loss of glycogen, with progression to fibrosis and hallmarks of energy deficiency, but shows ATP depletion only at end stage (Kotarsky et al., 2012; Purhonen et al., 2018). In a C57BL/6JCrl genetic background, the homozygotes survive much longer, up to 200 days, and they develop additional late-onset cardiomyopathy and mild cerebral astrogliosis (Rajendran et al., 2019). Intriguingly, further work in the original *Bcs1l^p.Ser78Gly^* strain that was developed on the C57BL/6JBomTac genetic background identified an mtDNA variant (*m.G14904A*) in the gene encoding the cytochrome b subunit of CIII (*mt-Cyb^p.Asp254Asn^*), which explains the short survival of the original strain (Purhonen et al., 2020). Thus, the two *Bcs1l^p.Ser78Gly^* mouse strains with different mtDNA backgrounds recapitulate not only GRACILE syndrome but also other CIII deficiencies with later-onset cardiomyopathy.

The *Bcs1l^p.Ser78Gly^* and *Bcs1l^p.Ser78Gly^*; *mt-Cyb^p.Asp254Asn^* mouse models have been used in several dietary and pharmacological interventional studies. The simplest dietary intervention, fasting, surprisingly revealed that the CIII-deficient mice largely maintain normal systemic lipid mobilization in response to a short fasting, despite their already severe loss of adipose tissue (Tomašić et al., 2020). High-fat low-carbohydrate (ketogenic) diet fed from weaning onwards for 3 weeks or 3 months had a beneficial effect on the liver disease, ameliorating fibrosis and other hallmarks without exacerbating the hypoglycemia (Purhonen et al., 2017). Conversely, a high-glucose diet had no beneficial effect, even though it normalized some plasma amino acid levels (Rajendran et al., 2016). *Bcs1l^p.Ser78Gly^* mice show decreased hepatic NAD^+^, possibly due to decreased *de novo* biosynthesis. Unexpectedly, feeding the mice with the NAD^+^ precursor nicotinamide riboside had no effect on hepatic NAD^+^, disease progression or hepatic mitochondrial function in this model, although this intervention had beneficial effects in a number of other mouse models of mitochondrial or other metabolic diseases (Pirinen et al., 2020; Khan et al., 2014; Purhonen et al., 2018).

Alternative oxidases (AOXs) are mitochondrial inner membrane proteins of plants and some lower animals. They transfer electrons directly from coenzyme Q to oxygen, thus bypassing CIII and CIV (Banerjee et al., 2021). To test whether AOX would be beneficial in *Bcs1l^p.Ser78Gly^* mice, in which CIII is non-functional, Rajendran et al. crossed them with transgenic mice broadly expressing *Ciona intestinalis* AOX. The survival of AOX-expressing homozygotes was almost tripled, from 200 to 600 days, owing to complete prevention of lethal cardiomyopathy. Kidney tubulopathy and cerebral astrogliosis were also ameliorated (Rajendran et al., 2019). This dramatic outcome suggested that CIII-linked metabolic pathways that are not significantly restored by AOX must play a major role in the pathogenesis (Banerjee et al., 2021). Interestingly, pyocyanin, a quinol-like molecule that can function as an electron carrier, was recently shown to have beneficial effects in cell and *Drosophila* models of CIII deficiency caused by mutations in the *Ttc19* assembly factor gene (Peruzzo et al., 2021).

GRACILE syndrome is a very severe disease with neonatal lethality and, as such, represents a robust phenotype for studies in experimental models. However, most other CIII deficiencies, including those caused by other *BCS1L* mutations, are less severe, meaning that the patients live with the disease for many years or even decades and need life-long treatment options. Mitochondrial diseases are largely impairments of energy metabolism, and, as discussed above, dietary modification has the potential to modify and alleviate some of the metabolic defects of the patients. Indeed, a ketogenic diet has shown beneficial effects in mitochondrial myopathy patients and in a patient with Björnstad syndrome, a mild form of CIII deficiency (Ahola et al., 2016; Marina et al., 2020). These discoveries suggest that there is potential for dietary modification to alleviate some symptoms in CIII deficiency patients with less-severe clinical picture, although GRACILE syndrome patients may be beyond such interventions due to their severe fetal-onset phenotype. The preclinical interventions in mouse models of GRACILE syndrome and other CIII deficiencies will undoubtedly continue to generate interesting data that will hopefully shape clinical trial design in the future.

#### IOSCA

IOSCA (OMIM 271245) is an autosomal-recessive neurodegenerative disease caused by variants in *TWNK* (Box 1). The vast majority of patients are homozygous for the founder variant, c.1523A>G, p.Tyr508Cys (NM_021830.5) (Nikali et al., 2005). The MAF of *TWNK* c.1523A>G is 93 times higher in Finns than in non-Finnish Europeans (Table 1, Fig. 2) (Karczewski et al., 2020). IOSCA manifests after 1 year of age, with progressive hearing loss, optic atrophy, cerebellar atrophy, ophthalmoplegia with strabismus, athetosis and ataxia, and degeneration of the peripheral sensory nerves by teenage (Koskinen et al., 1994; Lönnqvist et al., 2009). Epileptic seizures may develop after teenage and progress to severe epileptic encephalopathy. Most patients with IOSCA survive to adulthood, but a few individuals present with a severe form of the disease, including liver damage, during early childhood. Female patients also suffer from hypergonadotropic hypogonadism (Koskinen et al., 1994; Lönnqvist et al., 2009). IOSCA is an mtDNA depletion syndrome because of lowered mtDNA levels in the liver and of CI deficiency in large neurons (Hakonen et al., 2008). However, mtDNA depletion in IOSCA is rather mild considering its severe clinical manifestations, and the actual disease-causing molecular mechanisms remain unknown.

IOSCA knock-in mice with the homologous patient mutation replicate the key disease manifestations and show metabolic changes that suggest a nucleotide metabolic defect, making them a suitable model for testing metabolic interventions as treatment options (Nikkanen et al., 2016). Computational protein modeling suggested that the mutant TWNK consumes nucleotides even when not replicating DNA, which might be an underlying cause of mitochondrial nucleotide depletion (Nikkanen et al., 2016). This hypothesis remains to be tested.

#### LPI

LPI (OMIM 222700) is a rare autosomal-recessive disease with only a few hundred patients reported worldwide. Over 50 of them are from Finland, where the frequency is the highest in the world (1 in 60,000). In LPI, transport of the dibasic cationic amino acids lysine, arginine and ornithine is defective at the basolateral membrane of epithelial cells of the renal tubules and small intestine (Rajantie et al., 1980; Toivonen et al., 2013), and massive amounts of lysine and moderate amounts of arginine and ornithine are lost in the urine. The combination of clinical symptoms, increased urinary excretion and low plasma concentrations of cationic amino acids, especially lysine, is indicative of LPI.

LPI typically presents in infancy with recurrent vomiting and diarrhea, especially at weaning, episodes of stupor and loss of consciousness after a protein-rich meal, poor feeding, failure to thrive and muscular hypotonia. Most patients develop a protective aversion to high-protein foods. Reduced lysine uptake has a prominent role in the poor growth and the skeletal and immunological manifestations in LPI. Postprandial orotic aciduria (Box 1) is almost always seen in untreated patients. Hyperammonemia is best explained by functional deficiency of the arginine and ornithine intermediates in hepatocytes (Rajantie et al., 1983). The most severe complications include acute alveolar proteinosis (Parto et al., 1993) or multiorgan failure, hematologic abnormalities like normochromic or hypochromic anemia (Box 1), leukopenia, thrombocytopenia and erythroblastophagocytosis, a clinical presentation resembling the hemophagocytic lymphohistiocytosis and disturbed proximal tubular function with proteinuria, which often progresses to glomerular dysfunction and end-stage renal failure (Kärki et al., 2016; Nicolas et al., 2016; Tanner et al., 2007a,b). Hypercholesterolemia, hypertriglyceridemia and acute pancreatitis may occur. Pregnancies of patients with LPI can be complicated by toxemia or bleeding during delivery, but many are completed successfully without any major problems (Tanner et al., 2006).

LPI is caused by the pathogenic variants in *SLC7A7* (Box 1). About 70 different variants spread along the entire gene have been reported (Torrents et al., 1999; Sebastio, et al., 2011; Martinelli et al., 2020). Nearly all Finnish patients are homozygous for the founder variant, c.895-2A>T, p.Tyr299Ilefs*10 (NM_003982.4), which leads to premature translation termination. The MAF of *SLC7A7* c.895-2A>T is 65 times higher in Finns than in non-Finnish Europeans (Table 1, Fig. 2) (Karczewski et al., 2020).

The principal aims of LPI treatment are to prevent hyperammonemia and to provide a sufficient supply of protein and essential amino acids for normal metabolism and growth (Tanner et al., 2007a,b). Protein tolerance can be improved with supplementary low-dose citrulline, which is readily absorbed and partially converted to arginine and ornithine, all of which improve the function of the urea cycle. In patients with constantly highly elevated glutamine and glycine, treatment with sodium benzoate, sodium phenylbutyrate or glycerol phenylbutyrate helps to reduce the nitrogen load. A carefully titrated dose of L-lysine-HCl can elevate the plasma lysine concentrations to low-normal range without side effects. Growth hormone therapy has been used in several children (Niinikoski et al., 2011), and hypercholesterolemia has successfully been treated with statins (Tanner et al., 2010). Treatment of the immunological and bone marrow complications, including clinical hemophagocytic lymphohistiocytosis, is still experimental, but immunosuppressive drugs have had good responses in individual cases (Bader-Meunier et al., 2000). Bronchoalveolar lavage and steroid therapy have been effective in some alveolar proteinosis cases (Parto et al., 1994), and immunostimulating adjuvant therapy has been tried to mitigate the immunological abnormalities (Tanner et al., 2017). The first *in vivo* model for LPI, a tamoxifen-inducible *Slc7a7* KO mouse that recapitulates the human disease phenotype and responds to citrulline treatment, has emerged as a promising tool for designing future therapies, especially for the immunological complications of the disease (Bodoy et al., 2019).

### The most recent additions to the FDH

Fulfilling criteria defined by Norio (2003a), three new diseases have been included in the FDH since 2003: the motor neuron disease spinal muscular atrophy, Jokela type (SMAJ), a mild-to-moderate intellectual disability called frontotemporal pachygyria (CRADD) and phosphoenolpyruvate carboxykinase deficiency, cytosolic (PCKDC), which is a treatable inborn error of metabolism with recognizable biomarkers (Table 3). Owing to relatively recent identification of these diseases, availability of proper disease models and knowledge on pathological mechanisms for targeted treatment development is limited (Table 2).

**Table 3.**
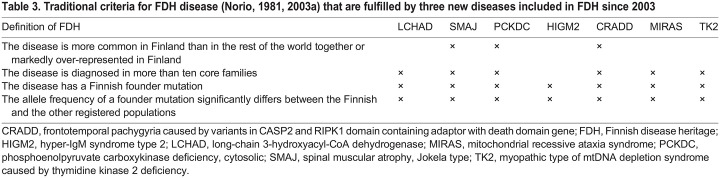
Traditional criteria for FDH disease (Norio, 1981, 2003a) that are fulfilled by three new diseases included in FDH since 2003

#### SMAJ

SMAJ (OMIM 615048), initially named late-onset spinal motor neuronopathy (LOSMoN), was described by Manu Jokela and colleagues in 2011 (Jokela et al., 2011). SMAJ is an adult-onset, progressive motor neuron disease in which muscle cramps are a typical initial manifestation, starting at ∼40 years of age. The cramps and fasciculations are followed by lower-limb-predominant muscle weakness, hyporeflexia and difficulties walking. Electromyography and muscle biopsy identify widespread neurogenic findings, and serum creatine kinase is elevated. Sensory defects may occur but are less common. A cure for SMAJ is not currently available, but medication may alleviate muscle cramps. Life expectancy of the patients is normal.

SMAJ is caused by a heterozygous *CHCHD10* (Box 1) variant, c.197G>T, p.Gly66Val (NM_213720.3), a founder mutation in the Finnish population with origin in North Karelia (Penttilä et al., 2015). Approximately 100 patients have been diagnosed, and 200-400 in total are predicted based on variant frequency of 0.0002 in the Finnish population (Penttilä et al., 2017; Karczewski et al., 2020). At the time of writing this Review, the variant has not been reported in other populations in the gnomAD database (Table 1, Fig. 2) (Karczewski et al., 2020). Other more-severe *CHCHD10* variants are a rare cause of dominant amyotrophic lateral sclerosis (ALS) and frontotemporal dementia (Bannwarth et al., 2014; Müller et al., 2014), and of mitochondrial myopathy (Ajroud-Driss et al., 2015), which has led to intense investigations of the underlying disease mechanisms.

Lack of CHCHD10 affects mitochondrial respiration, but its exact function is unknown (Straub et al., 2018; Harjuhaahto et al., 2020). Studies in cell and mouse models have shown that the ALS-linked variants cause CHCHD10 aggregation, which induces a mitochondrial integrated stress response and suggests a toxic gain-of-function effect (Anderson et al., 2019; Genin et al., 2019; Straub et al., 2021). Knock-in mouse models of the ALS-causing variant c.176C>T, p.Ser59Leu have myopathic and neurogenic changes, but also develop a lethal cardiomyopathy that is not observed in patients. The effects of the SMAJ variant c.197G>T, p.Gly66Val are likely to be different and less severe. Efforts to develop and characterize mouse models of SMAJ are ongoing, but no publications are available at the time of writing this Review. In addition, serum samples of SMAJ patients have been investigated to identify biomarkers for monitoring the effects of treatment trials in the future (Järvilehto et al., 2022).

#### Frontotemporal pachygyria (CRADD)

Frontotemporal pachygyria (OMIM 614499, MRT34) is an autosomal-recessive brain disease that typically presents in early childhood with delayed speech development and leads to mild-to-moderate intellectual disability. About half of the patients present with strabismus and aggressive behavior, and a subset present with abnormal electroencephalograms, epilepsy, distractibility, hyperactivity, attention deficit hyperactivity disorder (ADHD), macrocephaly or non-specific dysmorphic features such as a broad, prominent forehead. The hallmark of the disease is frontotemporal predominant pachygyria evident on brain MRI. Pachygyria is characterized by a reduced number of gyri, shallow sulci and mildly thickened cortex of 5-7 mm, whereas normal cortical thickness is 1-4.5 mm. Physical health of the patients is good, and life expectancy is normal. In some patients, intellectual disability has been so mild that it was diagnosed only during carrier screening of healthy siblings (Polla et al., 2019).

Frontotemporal pachygyria is caused by pathogenic variants in *CRADD* (Box 1). The *CRADD* founder variant, c.509G>A, p.Arg170His (NM_003805.5), is enriched in the Finnish population, with an allele frequency 50 times higher than in non-Finnish Europeans (Table 1, Fig. 2) (Karczewski et al., 2020). To date, a total of 17 families with 24 patients have been identified in Finland, mostly in Ostrobothnia and North-Eastern Finland (Kurki et al., 2019; Polla et al., 2019), all of them homozygous for the *CRADD* founder variant, c.509G>A, p.Arg170His, except one who was compound heterozygous for c.509G>A, p.Arg170His and c.2T>C, p.Met1? (Järvelä et al., 2021). Seven pathogenic *CRADD* variants have been detected to date, and the number of identified families outside Finland is less than 20 (Harel et al., 2017; Di Donato et al., 2016; Santos-Cortez et al., 2018).

*CRADD* is one of 19 known lissencephaly (Box 1) genes (Parrini et al., 2016). Pathogenic variants in CRADD abolish its ability to activate caspase-2, impairing neuronal apoptosis during brain development, a novel mechanism associated with cortical malformations (Di Donato et al., 2016). A KO mouse model of *Cradd* has megalencephaly and seizures, but no pachygyria (Table 2). Thus, the effect of CRADD on brain development differs in mouse and human, although some patients with biallelic pathogenic *CRADD* variants also have megalencephaly and epilepsy, meaning that researchers will need to develop alternative models to complement the KO mouse. Currently, there is no cure for the disease.

#### PCKDC

PCKDC (OMIM 261680) is another new addition to the FDH (Vieira et al., 2021). PCKDC is an inborn error of gluconeogenesis, leading to hypoglycemia in times of fasting or other inadequate carbohydrate intake (Table 2). The hypoglycemic episodes typically start before school age and become less frequent as the child grows. Hypoglycemia is possible in adulthood if predisposing factors such as fasting or alcohol consumption are present.

Phosphoenolpyruvate carboxykinase 1 (*PCK1*) variant c.925G>A, p.Gly309Arg (NM_002591.4) is prevalent in the Finnish population (Vieira et al., 2021, 2017), with an MAF that is 26 times higher in Finns than in non-Finnish Europeans (Table 1, Fig. 2) (Karczewski et al., 2020). To date, 22 Finnish individuals from 17 families have been identified to carry the homozygous founder variant, about double the number of previously described patients with other *PCK1* variants in the world. Additionally, two patients with compound heterozygous *PCK1* variants, c.925G>A, p.Gly309Arg and c.716C>T, p.Ser239Leu have been identified in Finland (Vieira et al., 2021). PCKDC cases have been diagnosed throughout Finland without any clear geographical clustering. Genealogical studies on the founder *PCK1* variant have not been performed yet; therefore, the origin of this mutation remains unclear.

In the Finnish cohort, nearly all the PCKDC patients presented with hypoketotic hypoglycemia, which can be recognized by laboratory screening tests. During hypoglycemic episodes, urine organic acids showed a recognizable abnormal pattern, with low or absent ketonuria, prominent fumaric aciduria, prominent adipic aciduria and, in most cases, prominent lactic aciduria (Vieira et al., 2017, 2021). Plasma amino acids typically showed low levels of glucogenic amino acids, such as serine and alanine. Proximal urea cycle dysfunction with elevated glutamine has been previously associated with PCKDC in patients presenting with acute liver failure (Santra et al., 2016), but, in the Finnish cohort, only a few patients showed mildly elevated glutamine levels, and none had elevated plasma ammonia levels (Vieira et al., 2021). PCKDC patients also often have mildly elevated liver transaminases and blood lactate levels during the hypoglycemic episodes. Liver transaminases and urine organic acids may take weeks or months to normalize. A recent report described a Hispanic PCKDC patient without documented hypoglycemia but presenting with growth failure (Oishi et al., 2021).

PCKDC patients can be symptom free with correct nutritional counseling. The patients should avoid fasting, and it is equally important to provide sufficient carbohydrate intake during times of inadequate nutrition. Thus, PCKDC is a treatable inborn error of metabolism with recognizable laboratory findings. As this disease has only recently been described, and the carrier frequency of *PCK1* variant c.925G>A is relatively high in the Finnish population, it is likely that more patients will be diagnosed in the future.

### Universal diseases with Finland-enriched pathogenic variants

Nowadays, new diseases and pathogenic variants are actively discovered worldwide, largely due to improved genetic and diagnostic tools. Detailed documentation of clinical manifestations of rare diseases has facilitated accurate identification and diagnostics of patients and identified many pathogenic variants of the same gene. One such example is hyper-IgM syndrome type 2 (HIGM2; OMIM 605258), which is a rare immunodeficiency caused by multiple different recessive variants in the *AICDA* gene (Table 2). Despite the MAF of the causative variant, c.416T>C, p.Met139Thr (NM_020661.2; rs200858797), being 72 times higher in Finns (0.001687) than in other populations of European origin (0.000024), as reported in gnomAD v.2.1.1 (Karczewski et al., 2020; Trotta et al., 2016), HIGM2 does not currently fulfill the traditional FDH criteria (Table 3). Similarly to HIGM2, long-chain 3-hydroxyacyl-CoA dehydrogenase (LCHAD) deficiency, mitochondrial recessive ataxia syndrome (MIRAS) and a myopathic type of mtDNA depletion syndrome (TK2 deficiency) are caused by ancestral variants more prevalent in the Finnish population and thus share many features with FDH diseases. However, these are not phenotypically restricted to Finland and are therefore also not considered part of the traditional FDH (Fig. 1).

#### LCHAD deficiency

**Fig. 3.**
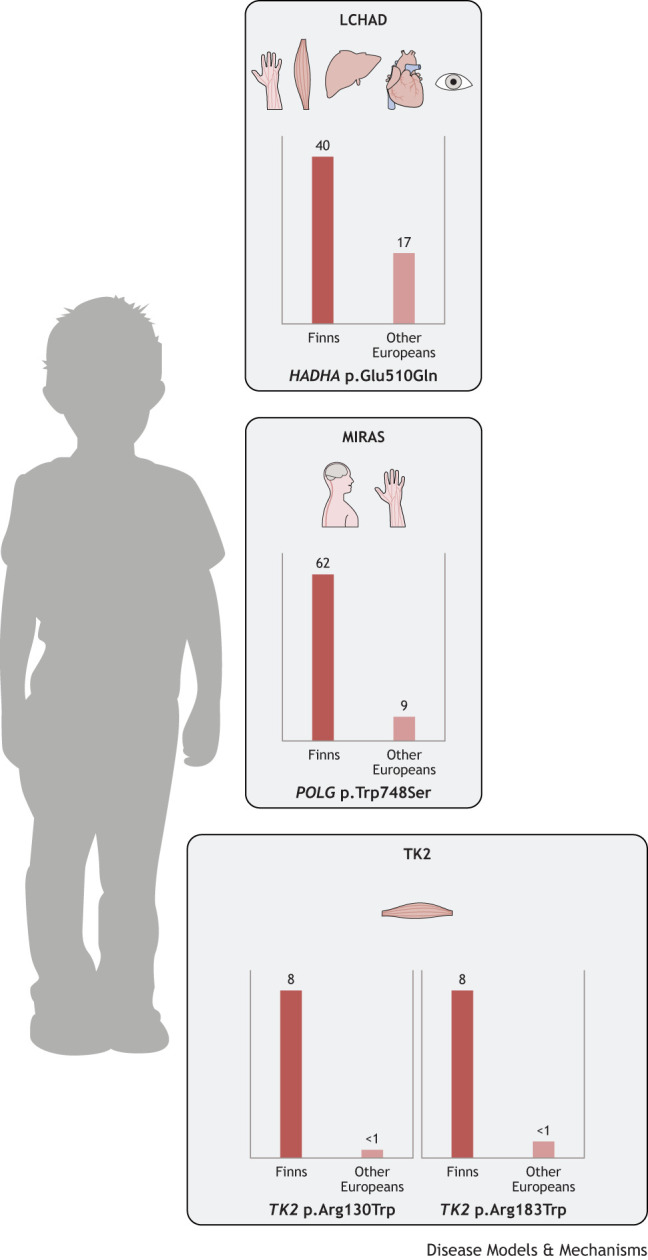
**Estimated carriers per 10,000 Finns and other Europeans of Finland-enriched pathogenic variants causing the ‘candidate’ FDH diseases long-chain 3-hydroxyacyl-CoA dehydrogenase deficiency (LCHAD), mitochondrial recessive ataxia syndrome (MIRAS) and myopathic type mtDNA depletion syndrome (TK2 deficiency)**. LCHAD is caused by a *HADHA* mutation that affects the peripheral nervous system, muscle, liver, heart and eyes; MIRAS is caused by *POLG* mutations that manifest as central and peripheral nervous system defects; and the two mutations causing TK2 deficiency affect the muscular system. Although the causative variants are enriched in the Finnish population, these diseases are not phenotypically restricted to Finland and are therefore not considered part of the traditional FDH. MAFs are based on gnomAD v2.1.1 (https://gnomad.broadinstitute.org/), accessed on 23 July 2022.

Patients with LCHAD deficiency (OMIM 609016) are typically healthy at birth, but, at 3-4 months of age, often triggered by an infection, they present with failure to thrive, hypotonia, lethargy, vomiting and metabolic crisis, including hypoglycemia, hyperammonemia, rhabdomyolysis, liver failure and cardiomyopathy. The pathogenesis of LCHAD deficiency was first described by Wanders et al. in 1990 (Wanders et al., 1990). It is caused by failure in the mitochondrial trifunctional protein (MTP; Box 1) complex. The two types of disorders of this complex are an isolated LCHAD deficiency (OMIM 609016) and an MTP deficiency (OMIM 609015). One of the causative variants of an isolated LCHAD deficiency is c.1528G>C, p.Glu510Gln (NM_000182.5; rs137852769) in the *HADHA* gene. This variant has been detected in many populations, but is more common in Finland than anywhere else, with a carrier frequency varying from 1:132 to 1:365 in rural and urban areas, respectively (Pastinen et al., 2001), compared to 1:680 in the Netherlands (den Boer et al., 2000) and 1:173 in Estonia (Joost et al., 2012). Its MAF is two times higher in Finns than in non-Finnish Europeans (Fig. 3) (Karczewski et al., 2020).

Newborn screening (NBS) has successfully provided early diagnosis for metabolic diseases, and hence therapeutic measures have been taken early enough to prevent irreversible damage. LCHAD deficiency can be reliably screened with dried blood spot acylcarnitine analysis, and LCHAD deficiency is included in the NBS panel in many countries, including Finland. However, contrary to many other metabolic diseases, the incidence of LCHAD deficiency has not increased in Finland since NBS started. The clinical presentation at 3-4 months of age is so typical that correct diagnosis could be accomplished without the acylcarnitine analysis. NBS has, however, brought children to medical services in a timely manner, avoiding long intensive care and improving prognosis compared to 20-30 years ago.

The current treatment is based on special diet with a significantly reduced fat intake (Immonen et al., 2016), but care must be taken that essential oils and fat-soluble vitamins are given in sufficient amount. Catabolism leads to the release of harmful fatty acids from adipose tissue into the circulation, and this should be avoided. The patients are fed regularly, and young ones receive continuous overnight feeds with carbohydrates and protein.

The treatment is very successful in preventing hypoglycemia and other acute complications, but patients are at risk of peripheral neuropathy or pigmentary retinopathy later in life (Uusimaa et al., 1997; Tyni et al., 1997). It seems, however, that the current treatment at least delays, if not abolishes, these complications (Immonen et al., 2016). Better understanding of the physiological consequences of metabolite accumulation in different tissues is important for improving the quality of life of LCHAD deficiency patients. For that reason, *in vivo* and *in vitro* models are being constantly developed, e.g. for LCHAD deficiency-associated retinopathy (Polinati et al., 2015).

#### MIRAS

In Finland, MIRAS (OMIM 607459) is caused by an ancestral mutation in the nuclear *POLG* (Box 1), which encodes the mtDNA polymerase. Even though the patients are typically homozygous for the ancestral MIRAS allele, the disease can manifest as (1) teenage-onset acute status epilepticus with valproate-induced hepatotoxicity; (2) early-adulthood-onset sensory polyneuropathy and spinocerebellar ataxia disease that may be accompanied by epilepsy; (3) middle-age-onset polyneuropathy-parkinsonism-ophthalmoplegia disease; or (4) sensory polyneuropathy only (Hakonen et al., 2005; Tzoulis et al., 2006; Uusimaa et al., 2008). Various psychiatric symptoms are often present, from depression and anxiety to paranoia, and cognitive decline may be part of the progressing disease. The cause of the variable phenotypes is unknown, but suggests the existence of major unidentified risk or protective factors.

As is typical for all POLG diseases, one recessive disease allele can carry several polymorphic variants, which may confuse diagnostic interpretation if segregation analysis is not performed. The Finnish MIRAS allele carries two (Eastern Finland) or three amino-acid-changing variants (Western Finland and Scandinavia). The disease-causing c.2243G>C, p.Trp748Ser (NM_002693.3) exists in combination with a common polymorphic variant c.3428A>G, p.Glu1143Gly (NM_002693.3) (Hakonen et al., 2005). However, an additional change, c.1491G>C, p.Gln497His (NM_002693.3; rs121918052), may exist in the same polypeptide with p.Trp748Ser and p.Glu1143Gly. Therefore, a heterozygous MIRAS allele carrier may carry three amino-acid-changing variants. *POLG* c.2243G>C, p.Trp748Ser is enriched in the Finnish population and has seven times higher MAF in Finns than in non-Finnish Europeans (Fig. 3) (Karczewski et al., 2020).

A typical brain imaging finding in MIRAS is bilateral symmetric lesions in the thalami and cerebellum (Rantamäki et al., 2001). Autopsy samples show respiratory chain deficiency in the large neurons, loss of cerebellar Purkinje cells and spongiotic changes in the above-mentioned brain regions (Rantamäki et al., 2001; Hakonen et al., 2005). Alongside in the brain, mtDNA depletion may occur in the muscle and liver. Multiple deletions of mtDNA can often be found in the muscle biopsy sample, but histological findings of respiratory chain deficiency, if present at all, are typically limited to a few abnormal muscle fibers.

Compound heterozygosity for the MIRAS allele with rare *POLG* variants causes an early-childhood-onset severe, progressive and fatal epileptic encephalopathy, Alpers-Huttenlocher syndrome (Naviaux and Nguyen, 2004). A second globally common variant, c.1399G>A, p.A467T, is often detected in combination with the MIRAS allele, causing a severe form of the disease (Tzoulis et al., 2006). However, this variant is not enriched in Finland, and the vast majority of the patients are homozygous for the p.W748S variant.

#### TK2 deficiency

Recessive variants in the nuclear-encoded thymidine kinase 2 (*TK2*; Box 1) cause a severe progressive depletion of mtDNA in skeletal muscle (OMIM 609560) (Garone et al., 2018). Two ancestral disease-causing variants, c.388C>T and c.547C>T (NM_004614.5), leading to p.Arg130Trp (rs281865493) and p.Arg183Trp (rs137886900), respectively, at conserved protein sites exist in Finland and occur as homozygous or compound heterozygous (Götz et al., 2008). The MAF of *TK2* c.388C>T is 21 times higher in Finns than in non-Finnish Europeans and that of *TK2* c.547C>T is ten times higher in Finns than in non-Finnish Europeans (Fig. 3) (Karczewski et al., 2020). The Finnish TK2 deficiency typically manifests in a previously healthy infant during the first months of life. The disease progresses rapidly to loss of skeletal muscle power and severe atrophy, mimicking spinal muscular atrophy. Histological examination of the skeletal muscle shows a large number of respiratory-chain-deficient fibers and severe muscle fibrosis and atrophy (Götz et al., 2008). Patients also have elevated blood levels of muscle-specific creatine kinase, which is not typical for other mitochondrial diseases.

An experimental treatment for TK2 deficiency based on deoxynucleoside supplementation has been developed and tested in mouse models expressing pathogenic *TK2* variants (Akman et al., 2008; Garone et al., 2014), as well as in patients. In patients, peroral deoxynucleoside therapy, under compassionate use, has significantly improved the condition in a subset of patients: some non-ambulatory patients gained the ability to walk, some were able to discontinue tube feeding, and most recipients experienced improvement in motor functional tests (Domínguez-González et al., 2019). However, if the disease had progressed to a stage at which mechanical ventilation was necessary, only one out of nine treated patients was able to resume independent breathing. In any case, this therapy trial gives a lot of hope for future ‘metabolic bypass’ therapies for TK2 deficiency and other FDH disorders: if an exact metabolic defect is identified, targeted metabolite treatment at an early stage of the disease can have dramatic effects.

## Justification for studying genetic and clinical data in Finland

Three reasons make the population of Finland attractive for combining genetic and health research. First, digitalized health records enable researchers to assign health care outcomes to study participants and thus create case-control settings across all types of outcomes. A particularly useful feature in Fennoscandic countries is that a unique personal identification number is assigned to each resident. That identification number can link an individual's information across all administrative registers, including digital health records. The identification number thus facilitates the accurate linking of all health record data to an individual, which helps to generate the health outcomes for the FinnGen project (Box 2). Digital health records, for example the hospital discharge registry, date back to the time when the register was started. In many cases, this therefore enables linkage of information to a participant decades prior to their sample donation for genetic analyses and does not rely on questionnaires. Data sources include drug purchases and prescriptions, hospital discharge diagnoses, procedure codes, the cancer registry and the cause of death register, offering a wealth of data for both cases and controls.

**Box 2. The FinnGen project.**

The FinnGen research project (https://www.finngen.fi/en/for_researchers), launched in 2017 and summarized in a recent preprint (Kurki et al., 2022 preprint), combines genome information with digital health care data from Finnish biobank participants. It is one of the first and largest public-private collaborative projects in this field with a goal to collect and analyze data from 500,000 participants. The project aims to improve human health through genetic research and ultimately identify new therapeutic targets and diagnostics for treating diseases. The FinnGen project brings together Finnish universities, research institutes, hospitals and hospital districts, biobanks and international pharmaceutical companies. Its collaborative nature ensures appropriate transparency, data security and ownership, and the project is based on the cooperation agreement between parties. University of Helsinki, Finland, is responsible for the FinnGen research project and is the official data controller of the study. The project has been evaluated by the Coordinating Ethics Committee of the Helsinki and Uusimaa Hospital District. The study complies with existing legislation and is funded by the public organization Business Finland and 12 international pharmaceutical companies. Each sample is pseudonymized so that individuals cannot be identified when biobank samples are used for FinnGen research. To ensure data security, data are not released but instead analyzed in a secure environment with controlled access rights.

Second, Finland's biobank legislation facilitates efficient sample collection by University Central hospitals in relevant clinical interest areas such as neurology, gastroenterology, rheumatology, pulmonary diseases, cardiometabolic diseases, oncology and ophthalmology. The FinnGen samples are derived from two separate sources: the legacy biobank collections, meaning old clinical sample collections and health surveys that had been transferred to biobanks and had either DNA material or genome-wide genotype data available, and through recruitment of new participants. In August 2021, the number of samples available was 496,200, out of which 356,077 had passed quality control and had genotype and phenotype data available for analysis.

Third, the population history that has generated the unique genetic make-up in Finns and caused the increase in the frequency of the FDH variants. This phenomenon is not limited to monogenic disease, though, and has affected the entire genome of Finns, impacting the frequency of haplotypes. Thus, this particular population history has also affected common complex diseases, as was demonstrated in the recent preprint by Kurki et al. (2022). Previously, Sun et al. (2022) studied protein-coding variation associations across 744 traits comparing data from the UK Biobank and the FinnGen project. They showed that, for numerous complex disease-associated variants, there are over fourfold allele frequency differences between the UK and Finland, underscoring population differences. There are also examples where the allele frequency of a certain variant is much smaller in Finns than in an admixed European sample, and vice versa, while there are variants enriched in Finns that occur at much lower frequencies in the European population. The preprint by Heyne et al. (2021) suggests that Finnish genomes harbor fewer detectable variants for recessively inherited disease variants than non-Finnish Europeans, but those that are detected have larger MAFs. Therefore, alongside the FDH, Finnish genomes are also enriched for variants linked to more genetically complex diseases, which can facilitate discoveries in the common complex disease spectrum with implications beyond this single population.

### Collection of genomic information

To facilitate the most efficient discovery of genetic, particularly coding, variation enriched in Finland, a tailored genotyping array was generated for the FinnGen project. The array was designed to include 116,402 exonic variants detected in the exomes of ∼20,000 Finnish individuals, together with an imputation backbone of 540,008 variants optimized for the Finnish population. Furthermore, the array was extended to HLA-specific variants, ClinVar database (https://www.ncbi.nlm.nih.gov/clinvar/) pathogenic variants and other custom content, totaling 736,145 variants. To maximize the accuracy of imputation, FinnGen also uses a population-specific imputation reference panel of over 8000 deep-sequenced participants. FinnGen operates in 6-month data release cycles (https://finngen.gitbook.io/documentation/releases), ensuring that the expanding dataset remains up to date and accessible.

### Genotype–phenotype discoveries

Since its establishment in 2017, the FinnGen project has already significantly contributed to numerous discoveries of genotype–phenotype associations, which have been validated in other populations using data from UK Biobank (www.ukbiobank.ac.uk) and/or Estonian Biobank (https://genomics.ut.ee/en/content/estonian-biobank). As an example, FinnGen spearheaded the discovery of two novel genetic loci, *DSC1* and *SERPINB7*, predisposing to atopic dermatitis (Sliz et al., 2021), and of cervical-cancer-predisposing variants in *PAX8*, *CLPTM1L* and HLA genes (Bowden et al., 2021). Further examples of genotype associations with common complex traits include variants in *MYO10* and *CHEK2*, predisposing to polycystic ovary syndrome (Tyrmi et al., 2021), and a missense variant in *SPDL1* associated with idiopathic pulmonary fibrosis (Dhindsa et al., 2021). Higher allele frequencies in Finns significantly increased the power to discover these associations, which are much less frequent in other populations and would thus have required much larger sample sizes for the initial discovery in non-Finnish populations. Even though these associations have already been validated in an independent population, replication in other populations will be of high value in the future.

The recruitment of participants is bound to generate some bias towards certain selected outcomes. In FinnGen, the legacy collections described above were created for heterogeneous purposes. The numbers of cases depend on the rate of recruitment, and, for areas of interest, the frequency of cases is likely somewhat higher than the population average. For other outcomes, the effect of recruitment on the case frequency is unknown. It is likely that FinnGen has selection bias against severe, early-onset diseases such as many of the FDH diseases, and those that render the carriers incapable to participate. However, as the medical records trace back decades, FinnGen contains a wealth of data to study vast numbers of different outcomes and, together with the Sequencing Initiative Suomi (SISu) project (http://www.sisuproject.fi/), a search engine for sequence variants in Finns, provides a national reference genome to identify novel, Finland-enriched pathogenic variants. As FinnGen aims to genotype roughly 10% of the Finnish population, the sample size allows the study of genetic diseases with low frequencies in the population.

## Future perspectives

The genetic landscape of Finns has historically been heavily influenced by population isolates within the country, leading to enrichment of certain variants and under-representation of others, a finding that is supported by FinnGen data. Recessive, disease-causing variants have remained present in many areas, although their incidence is decreasing. Inhabitation preference to an urban lifestyle is likely to influence the future of FDH and shape the disease prevalence and spectrum in Finland and elsewhere. All these factors are also affected by increased immigration (https://www.stat.fi/index_en.html), which leads to variant crossover with possible effects on disease manifestation and/or severity. Moreover, immigration also increases the frequency of diseases that are common elsewhere but have previously been ultra-rare in Finland. An example of the latter is already being seen in genetic counseling clinics, where clear increment in people originating from countries in which hemoglobinopathies are common (https://emn.fi/wp-content/uploads/2022/08/EMN_tilastokatsaus_2021_netti_EN_FINAL.pdf) has led to the introduction of thalassemia and sickle cell anemia (Vives et al., 2010).

An interesting counterpart to this changing genetic landscape is a diminished frequency of certain FDH disorders, like AGU, which partly derives from the improved availability of genetic counseling and prenatal diagnostics, as well as preimplantation diagnostics and screening. These offer personalized possibilities for family planning, which is especially important not only for families at risk of fetal and neonatal forms of FDH that lead to death of the fetus or neonate, such as hydrolethalus syndrome 1, lethal congenital contracture syndrome 1 and lethal arthrogryposis with anterior horn cell disease, but also for families at risk of severe progressive pediatric-onset diseases like AGU, ceroid lipofuscinosis, neuronal, 1 (CLN1), GRACILE syndrome, IOSCA and many others. Development of non-invasive genetic screens enabling analysis of cell-free fetal DNA in maternal plasma may complement current diagnostic methods (Pös et al., 2019). In addition, increasing clinical genomic approaches, together with biobank-data-based research, could facilitate efficient carrier detection of FDH diseases.

The geographical restriction of the FDH is also changing. This is partly because of the previous work done by the research and clinical communities and partly because of the technical advances in genome sequencing. Active dissemination of the detailed disease descriptions and of the causative variant of a given disorder has facilitated identification of FDH-like disorders worldwide, as exemplified by AGU, for which more than 30 *AGA* variants have been identified globally (Goodspeed et al., 2021). A feature typical of non-Finnish FDH-like disorders is that the causative variant differs from the major Finnish variant, and its identification has been facilitated by the availability of NBS, molecular genetic testing or next-generation sequencing (Opladen et al., 2014; Sainio et al., 2021). In the 21st century, three new diseases of FDH have been identified and characterized. In the future, the number of cases with FDH-like diseases is likely to increase worldwide, and new pathogenic variants could be introduced to the Finnish population that might also prompt re-evaluation of the FDH criteria. It is important to maintain active research that aims to catalogue novel disease-causing variants and to understand their molecular and cellular pathomechanisms. These collective endeavors will facilitate the diagnosis of rare diseases and provide grounds for understanding of common diseases as well.
